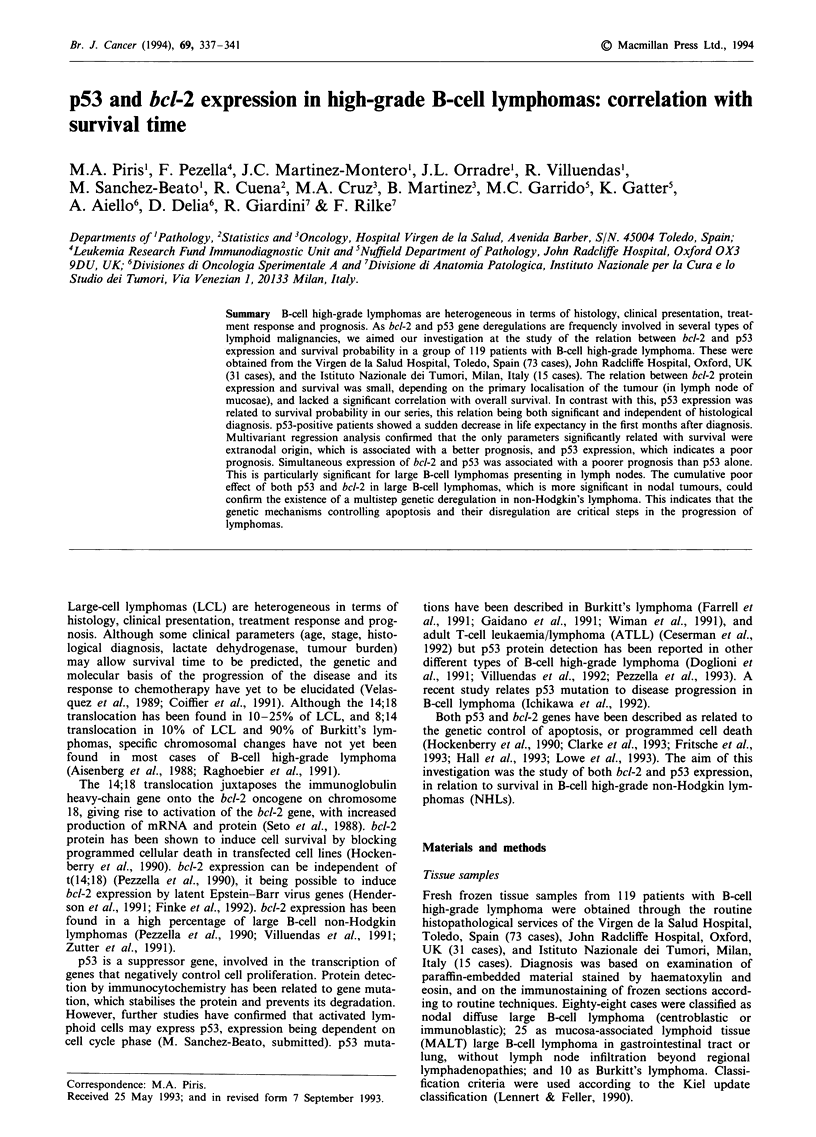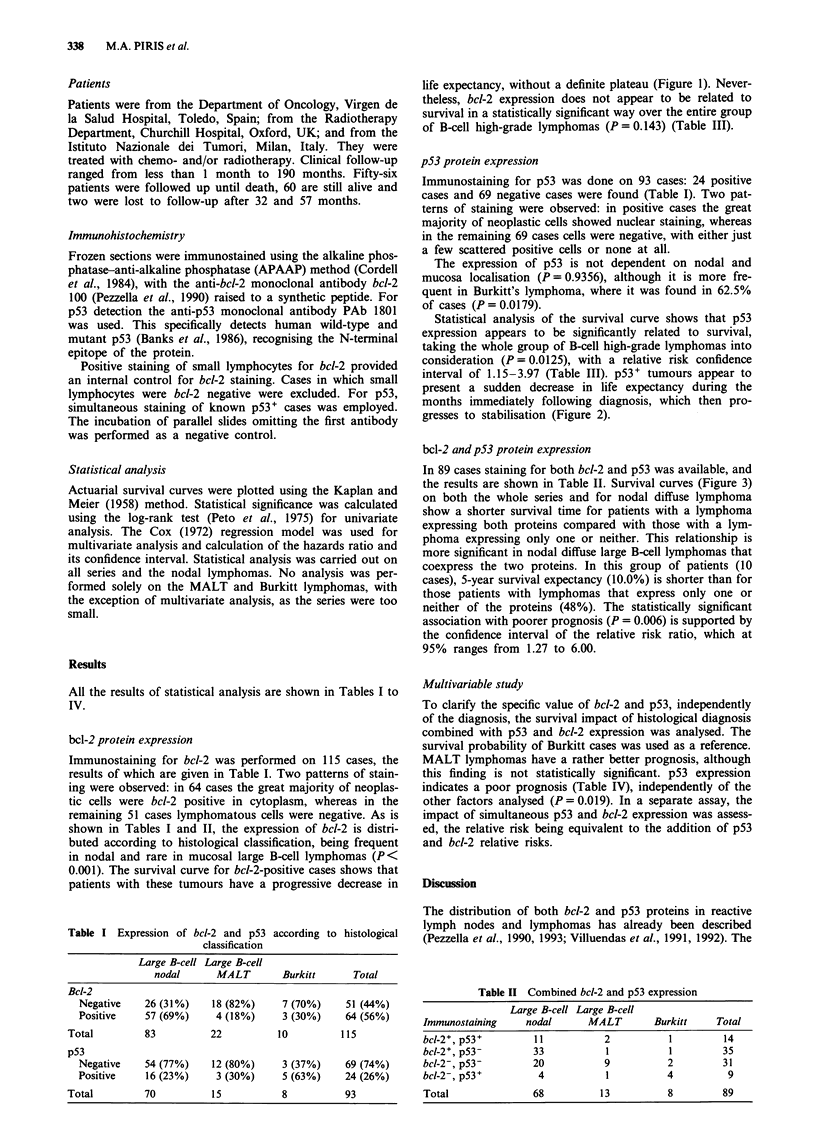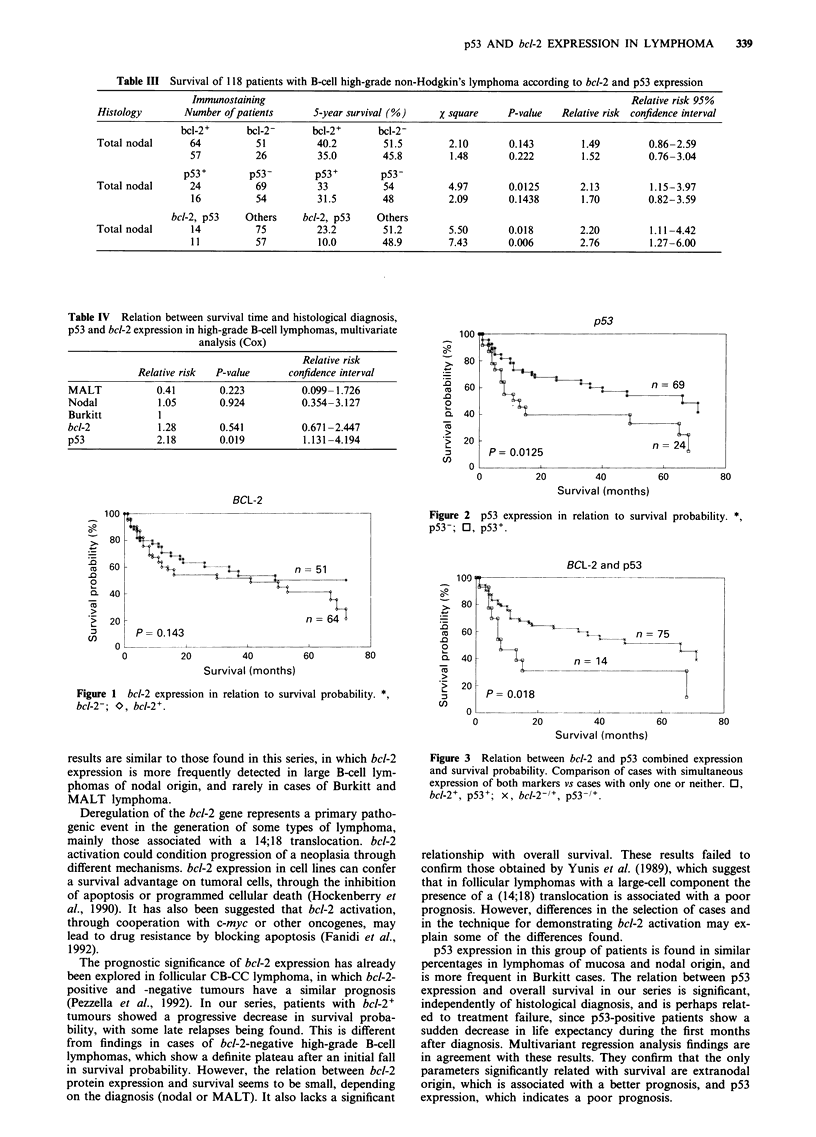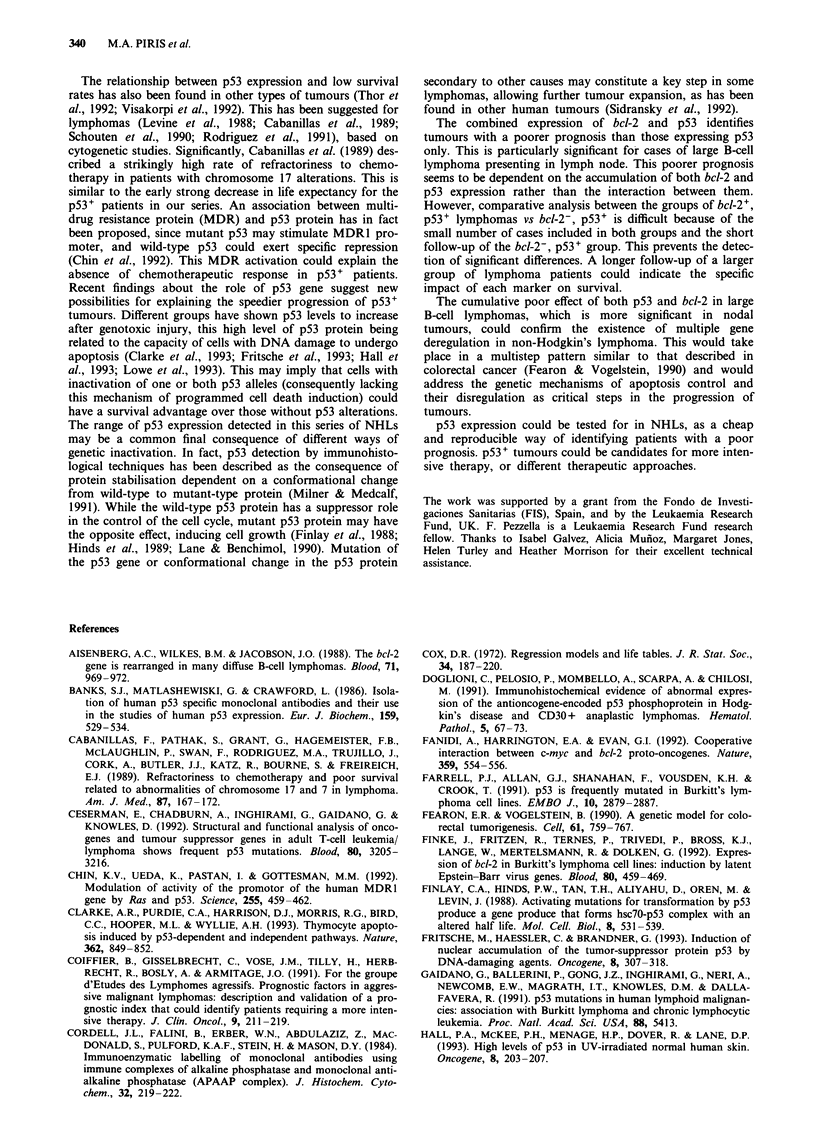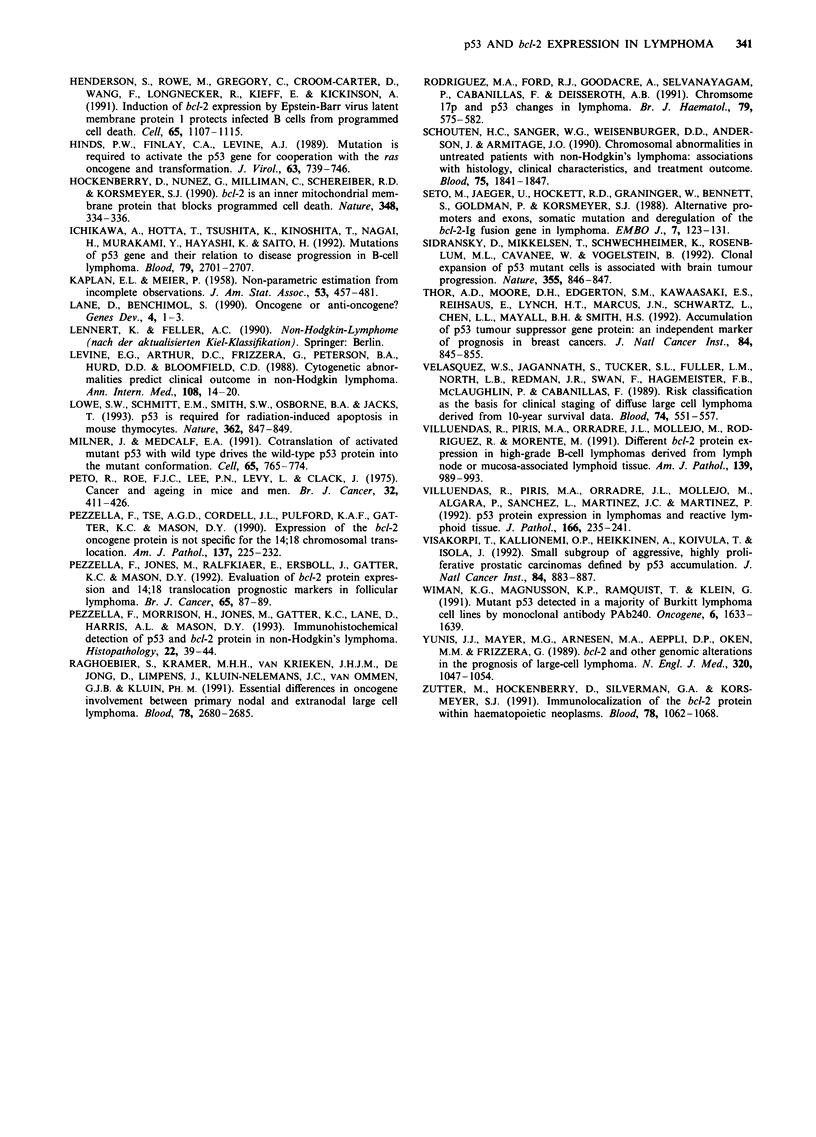# p53 and bcl-2 expression in high-grade B-cell lymphomas: correlation with survival time.

**DOI:** 10.1038/bjc.1994.61

**Published:** 1994-02

**Authors:** M. A. Piris, F. Pezzella, J. C. Martinez-Montero, J. L. Orradre, R. Villuendas, M. Sanchez-Beato, R. Cuena, M. A. Cruz, B. Martinez, F. ]. Pezella F [corrected to Pezzella

**Affiliations:** Department of Pathology, Hospital Virgen de la Salud, Toledo, Spain.

## Abstract

B-cell high-grade lymphomas are heterogeneous in terms of histology, clinical presentation, treatment response and prognosis. As bcl-2 and p53 gene deregulations are frequently involved in several types of lymphoid malignancies, we aimed our investigation at the study of the relation between bcl-2 and p53 expression and survival probability in a group of 119 patients with B-cell high-grade lymphoma. These were obtained from the Virgen de la Salud Hospital, Toledo, Spain (73 cases), John Radcliffe Hospital, Oxford, UK (31 cases), and the Istituto Nazionale dei Tumori, Milan, Italy (15 cases). The relation between bcl-2 protein expression and survival was small, depending on the primary localisation of the tumour (in lymph node of mucosae), and lacked a significant correlation with overall survival. In contrast with this, p53 expression was related to survival probability in our series, this relation being both significant and independent of histological diagnosis. p53-positive patients showed a sudden decrease in life expectancy in the first months after diagnosis. Multivariant regression analysis confirmed that the only parameters significantly related with survival were extranodal origin, which is associated with a better prognosis, and p53 expression, which indicates a poor prognosis. Simultaneous expression of bcl-2 and p53 was associated with a poorer prognosis than p53 alone. This is particularly significant for large B-cell lymphomas presenting in lymph nodes. The cumulative poor effect of both p53 and bcl-2 in large B-cell lymphomas, which is more significant in nodal tumours, could confirm the existence of a multistep genetic deregulation in non-Hodgkin's lymphoma. This indicates that the genetic mechanisms controlling apoptosis and their disregulation are critical steps in the progression of lymphomas.


Br. J. Cancer (1994), 69, 337-341 © Macmillan Press Ltd., 1994

### p53 and *bcl*-2 expression in high-grade B-cell lymphomas: correlation with survival time

M.A. Piris[1], F. Pezella[4], J.C. Martinez-Montero[1], J.L. Orradre[1], R. Villuendas[1], M. Sanchez-Beato[1], R. Cuena[2], M.A. Cruz[3], B. Martinez[3], M.C. Garrido[5], K. Gatter[5], A. Aiello[6], D. Delia[6], R. Giardini[7] & F. Rilke[7]

*Departments of [1]Pathology, [2]Statistics and [3]Oncology, Hospital Virgen de la Salud, Avenida Barber, S/N. 45004 Toledo, Spain; [4]Leukemia Research Fund Immunodiagnostic Unit and [5]Nuffield Department of Pathology, John Radcliffe Hospital, Oxford OX3 9DU, UK; [6]Divisiones di Oncologia Sperimentale A and [7]Divisione di Anatomia Patologica, Instituto Nazionale per la Cura e lo Studio dei Tumori, Via Venezian 1, 20133 Milan, Italy.*

**Summary** B-cell high-grade lymphomas are heterogeneous in terms of histology, clinical presentation, treatment response and prognosis. As *bcl*-2 and p53 gene deregulations are frequenly involved in several types of lymphoid malignancies, we aimed our investigation at the study of the relation between *bcl*-2 and p53 expression and survival probability in a group of 119 patients with B-cell high-grade lymphoma. These were obtained from the Virgen de la Salud Hospital, Toledo, Spain (73 cases), John Radcliffe Hospital, Oxford, UK (31 cases), and the Istituto Nazionale dei Tumori, Milan, Italy (15 cases). The relation between *bcl*-2 protein expression and survival was small, depending on the primary localisation of the tumour (in lymph node of mucosae), and lacked a significant correlation with overall survival. In contrast with this, p53 expression was related to survival probability in our series, this relation being both significant and independent of histological diagnosis. p53-positive patients showed a sudden decrease in life expectancy in the first months after diagnosis. Multivariant regression analysis confirmed that the only parameters significantly related with survival were extranodal origin, which is associated with a better prognosis, and p53 expression, which indicates a poor prognosis. Simultaneous expression of *bcl*-2 and p53 was associated with a poorer prognosis than p53 alone. This is particularly significant for large B-cell lymphomas presenting in lymph nodes. The cumulative poor effect of both p53 and *bcl*-2 in large B-cell lymphomas, which is more significant in nodal tumours, could confirm the existence of a multistep genetic deregulation in non-Hodgkin's lymphoma. This indicates that the genetic mechanisms controlling apoptosis and their disregulation are critical steps in the progression of lymphomas.

Large-cell lymphomas (LCL) are heterogeneous in terms of histology, clinical presentation, treatment response and prognosis. Although some clinical parameters (age, stage, histological diagnosis, lactate dehydrogenase, tumour burden) may allow survival time to be predicted, the genetic and molecular basis of the progression of the disease and its response to chemotherapy have yet to be elucidated (Velasquez *et al.*, 1989; Coiffier *et al.*, 1991). Although the 14;18 translocation has been found in 10-25% of LCL, and 8;14 translocation in 10% of LCL and 90% of Burkitt's lymphomas, specific chromosomal changes have not yet been found in most cases of B-cell high-grade lymphoma (Aisenberg *et al.*, 1988; Raghoebier *et al.*, 1991).

The 14;18 translocation juxtaposes the immunoglobulin heavy-chain gene onto the *bcl*-2 oncogene on chromosome 18, giving rise to activation of the *bcl*-2 gene, with increased production of mRNA and protein (Seto *et al.*, 1988). *bcl*-2 protein has been shown to induce cell survival by blocking programmed cellular death in transfected cell lines (Hockenberry *et al.*, 1990). *bcl*-2 expression can be independent of t(14;18) (Pezzella *et al.*, 1990), it being possible to induce *bcl*-2 expression by latent Epstein-Barr virus genes (Henderson *et al.*, 1991; Finke *et al.*, 1992). *bcl*-2 expression has been found in a high percentage of large B-cell non-Hodgkin lymphomas (Pezzella *et al.*, 1990; Villuendas *et al.*, 1991; Zutter *et al.*, 1991).

p53 is a suppressor gene, involved in the transcription of genes that negatively control cell proliferation. Protein detection by immunocytochemistry has been related to gene mutation, which stabilises the protein and prevents its degradation. However, further studies have confirmed that activated lymphoid cells may express p53, expression being dependent on cell cycle phase (M. Sanchez-Beato, submitted). p53 mutations have been described in Burkitt's lymphoma (Farrell *et al.*, 1991; Gaidano *et al.*, 1991; Wiman *et al.*, 1991), and adult T-cell leukaemia/lymphoma (ATLL) (Ceserman *et al.*, 1992) but p53 protein detection has been reported in other different types of B-cell high-grade lymphoma (Doglioni *et al.*, 1991; Villuendas *et al.*, 1992; Pezzella *et al.*, 1993). A recent study relates p53 mutation to disease progression in B-cell lymphoma (Ichikawa *et al.*, 1992).

Both p53 and *bcl*-2 genes have been described as related to the genetic control of apoptosis, or programmed cell death (Hockenberry *et al.*, 1990; Clarke *et al.*, 1993; Fritsche *et al.*, 1993; Hall *et al.*, 1993; Lowe *et al.*, 1993). The aim of this investigation was the study of both *bcl*-2 and p53 expression, in relation to survival in B-cell high-grade non-Hodgkin lymphomas (NHLs).

### Materials and methods

### *Tissue samples*

Fresh frozen tissue samples from 119 patients with B-cell high-grade lymphoma were obtained through the routine histopathological services of the Virgen de la Salud Hospital, Toledo, Spain (73 cases), John Radcliffe Hospital, Oxford, UK (31 cases), and Istituto Nazionale dei Tumori, Milan, Italy (15 cases). Diagnosis was based on examination of paraffin-embedded material stained by haematoxylin and eosin, and on the immunostaining of frozen sections according to routine techniques. Eighty-eight cases were classified as nodal diffuse large B-cell lymphoma (centroblastic or immunoblastic); 25 as mucosa-associated lymphoid tissue (MALT) large B-cell lymphoma in gastrointestinal tract or lung, without lymph node infiltration beyond regional lymphadenopathies; and 10 as Burkitt's lymphoma. Classification criteria were used according to the Kiel update classification (Lennert & Feller, 1990).

Correspondence: M.A. Piris.
Received 25 May 1993; and in revised form 7 September 1993.

*Patients*

Patients were from the Department of Oncology, Virgen de la Salud Hospital, Toledo, Spain; from the Radiotherapy Department, Churchill Hospital, Oxford, UK; and from the Istituto Nazionale dei Tumori, Milan, Italy. They were treated with chemo- and/or radiotherapy. Clinical follow-up ranged from less than 1 month to 190 months. Fifty-six patients were followed up until death, 60 are still alive and two were lost to follow-up after 32 and 57 months.

*Immunohistochemistry*

Frozen sections were immunostained using the alkaline phosphatase–anti-alkaline phosphatase (APAAP) method (Cordell *et al.*, 1984), with the anti-*bcl*-2 monoclonal antibody *bcl*-2 100 (Pezzella *et al.*, 1990) raised to a synthetic peptide. For p53 detection the anti-p53 monoclonal antibody PAb 1801 was used. This specifically detects human wild-type and mutant p53 (Banks *et al.*, 1986), recognising the N-terminal epitope of the protein.

Positive staining of small lymphocytes for *bcl*-2 provided an internal control for *bcl*-2 staining. Cases in which small lymphocytes were *bcl*-2 negative were excluded. For p53, simultaneous staining of known $p53^+$ cases was employed. The incubation of parallel slides omitting the first antibody was performed as a negative control.

*Statistical analysis*

Actuarial survival curves were plotted using the Kaplan and Meier (1958) method. Statistical significance was calculated using the log-rank test (Peto *et al.*, 1975) for univariate analysis. The Cox (1972) regression model was used for multivariate analysis and calculation of the hazards ratio and its confidence interval. Statistical analysis was carried out on all series and the nodal lymphomas. No analysis was performed solely on the MALT and Burkitt lymphomas, with the exception of multivariate analysis, as the series were too small.

### Results

All the results of statistical analysis are shown in Tables I to IV.

bcl*-2 protein expression*

Immunostaining for *bcl*-2 was performed on 115 cases, the results of which are given in Table I. Two patterns of staining were observed: in 64 cases the great majority of neoplastic cells were *bcl*-2 positive in cytoplasm, whereas in the remaining 51 cases lymphomatous cells were negative. As is shown in Tables I and II, the expression of *bcl*-2 is distributed according to histological classification, being frequent in nodal and rare in mucosal large B-cell lymphomas ($P<0.001$). The survival curve for *bcl*-2-positive cases shows that patients with these tumours have a progressive decrease in life expectancy, without a definite plateau (Figure 1). Nevertheless, *bcl*-2 expression does not appear to be related to survival in a statistically significant way over the entire group of B-cell high-grade lymphomas ($P = 0.143$) (Table III).

*p53 protein expression*

Immunostaining for p53 was done on 93 cases: 24 positive cases and 69 negative cases were found (Table I). Two patterns of staining were observed: in positive cases the great majority of neoplastic cells showed nuclear staining, whereas in the remaining 69 cases cells were negative, with either just a few scattered positive cells or none at all.

The expression of p53 is not dependent on nodal and mucosa localisation ($P = 0.9356$), although it is more frequent in Burkitt's lymphoma, where it was found in 62.5% of cases ($P = 0.0179$).

Statistical analysis of the survival curve shows that p53 expression appears to be significantly related to survival, taking the whole group of B-cell high-grade lymphomas into consideration ($P = 0.0125$), with a relative risk confidence interval of 1.15–3.97 (Table III). $p53^+$ tumours appear to present a sudden decrease in life expectancy during the months immediately following diagnosis, which then progresses to stabilisation (Figure 2).

bcl*-2 and p53 protein expression*

In 89 cases staining for both *bcl*-2 and p53 was available, and the results are shown in Table II. Survival curves (Figure 3) on both the whole series and for nodal diffuse lymphoma show a shorter survival time for patients with a lymphoma expressing both proteins compared with those with a lymphoma expressing only one or neither. This relationship is more significant in nodal diffuse large B-cell lymphomas that coexpress the two proteins. In this group of patients (10 cases), 5-year survival expectancy (10.0%) is shorter than for those patients with lymphomas that express only one or neither of the proteins (48%). The statistically significant association with poorer prognosis ($P = 0.006$) is supported by the confidence interval of the relative risk ratio, which at 95% ranges from 1.27 to 6.00.

*Multivariable study*

To clarify the specific value of *bcl*-2 and p53, independently of the diagnosis, the survival impact of histological diagnosis combined with p53 and *bcl*-2 expression was analysed. The survival probability of Burkitt cases was used as a reference. MALT lymphomas have a rather better prognosis, although this finding is not statistically significant. p53 expression indicates a poor prognosis (Table IV), independently of the other factors analysed ($P = 0.019$). In a separate assay, the impact of simultaneous p53 and *bcl*-2 expression was assessed, the relative risk being equivalent to the addition of p53 and *bcl*-2 relative risks.

### Discussion

The distribution of both *bcl*-2 and p53 proteins in reactive lymph nodes and lymphomas has already been described (Pezzella *et al.*, 1990, 1993; Villuendas *et al.*, 1991, 1992). The

**Table I** Expression of *bcl*-2 and p53 according to histological classification

| | *Large B-cell nodal* | *Large B-cell MALT* | *Burkitt* | *Total* |
|---|---|---|---|---|
| *Bcl-2* | | | | |
| Negative | 26 (31%) | 18 (82%) | 7 (70%) | 51 (44%) |
| Positive | 57 (69%) | 4 (18%) | 3 (30%) | 64 (56%) |
| Total | 83 | 22 | 10 | 115 |
| p53 | | | | |
| Negative | 54 (77%) | 12 (80%) | 3 (37%) | 69 (74%) |
| Positive | 16 (23%) | 3 (30%) | 5 (63%) | 24 (26%) |
| Total | 70 | 15 | 8 | 93 |

**Table II** Combined *bcl*-2 and p53 expression

| *Immunostaining* | *Large B-cell nodal* | *Large B-cell MALT* | *Burkitt* | *Total* |
|---|---|---|---|---|
| *bcl*-$2^+$, $p53^+$ | 11 | 2 | 1 | 14 |
| *bcl*-$2^+$, $p53^-$ | 33 | 1 | 1 | 35 |
| *bcl*-$2^-$, $p53^-$ | 20 | 9 | 2 | 31 |
| *bcl*-$2^-$, $p53^+$ | 4 | 1 | 4 | 9 |
| Total | 68 | 13 | 8 | 89 |

**Table III** Survival of 118 patients with B-cell high-grade non-Hodgkin's lymphoma according to *bcl*-2 and p53 expression

| *Histology* | *Immunostaining Number of patients* | | *5-year survival (%)* | | *χ square* | *P-value* | *Relative risk* | *Relative risk 95% confidence interval* |
|---|---|---|---|---|---|---|---|---|
| | bcl-2$^+$ | bcl-2$^-$ | bcl-2$^+$ | bcl-2$^-$ | | | | |
| Total nodal | 64 | 51 | 40.2 | 51.5 | 2.10 | 0.143 | 1.49 | 0.86-2.59 |
| | 57 | 26 | 35.0 | 45.8 | 1.48 | 0.222 | 1.52 | 0.76-3.04 |
| | p53$^+$ | p53$^-$ | p53$^+$ | p53$^-$ | | | | |
| Total nodal | 24 | 69 | 33 | 54 | 4.97 | 0.0125 | 2.13 | 1.15-3.97 |
| | 16 | 54 | 31.5 | 48 | 2.09 | 0.1438 | 1.70 | 0.82-3.59 |
| | *bcl*-2, p53 | Others | *bcl*-2, p53 | Others | | | | |
| Total nodal | 14 | 75 | 23.2 | 51.2 | 5.50 | 0.018 | 2.20 | 1.11-4.42 |
| | 11 | 57 | 10.0 | 48.9 | 7.43 | 0.006 | 2.76 | 1.27-6.00 |

**Table IV** Relation between survival time and histological diagnosis, p53 and *bcl*-2 expression in high-grade B-cell lymphomas, multivariate analysis (Cox)

| | *Relative risk* | *P-value* | *Relative risk confidence interval* |
|---|---|---|---|
| MALT | 0.41 | 0.223 | 0.099-1.726 |
| Nodal | 1.05 | 0.924 | 0.354-3.127 |
| Burkitt | 1 | | |
| *bcl*-2 | 1.28 | 0.541 | 0.671-2.447 |
| p53 | 2.18 | 0.019 | 1.131-4.194 |

**Figure 1** *bcl*-2 expression in relation to survival probability. *, *bcl*-2$^-$; ◇, *bcl*-2$^+$.

**Figure 2** p53 expression in relation to survival probability. *, p53$^-$; □, p53$^+$.

**Figure 3** Relation between *bcl*-2 and p53 combined expression and survival probability. Comparison of cases with simultaneous expression of both markers *vs* cases with only one or neither. □, *bcl*-2$^+$, p53$^+$; ×, *bcl*-2$^{-/+}$, p53$^{-/+}$.

results are similar to those found in this series, in which *bcl*-2 expression is more frequently detected in large B-cell lymphomas of nodal origin, and rarely in cases of Burkitt and MALT lymphoma.

Deregulation of the *bcl*-2 gene represents a primary pathogenic event in the generation of some types of lymphoma, mainly those associated with a 14;18 translocation. *bcl*-2 activation could condition progression of a neoplasia through different mechanisms. *bcl*-2 expression in cell lines can confer a survival advantage on tumoral cells, through the inhibition of apoptosis or programmed cellular death (Hockenberry *et al.*, 1990). It has also been suggested that *bcl*-2 activation, through cooperation with c-*myc* or other oncogenes, may lead to drug resistance by blocking apoptosis (Fanidi *et al.*, 1992).

The prognostic significance of *bcl*-2 expression has already been explored in follicular CB-CC lymphoma, in which *bcl*-2-positive and -negative tumours have a similar prognosis (Pezzella *et al.*, 1992). In our series, patients with *bcl*-2$^+$ tumours showed a progressive decrease in survival probability, with some late relapses being found. This is different from findings in cases of *bcl*-2-negative high-grade B-cell lymphomas, which show a definite plateau after an initial fall in survival probability. However, the relation between *bcl*-2 protein expression and survival seems to be small, depending on the diagnosis (nodal or MALT). It also lacks a significant relationship with overall survival. These results failed to confirm those obtained by Yunis *et al.* (1989), which suggest that in follicular lymphomas with a large-cell component the presence of a (14;18) translocation is associated with a poor prognosis. However, differences in the selection of cases and in the technique for demonstrating *bcl*-2 activation may explain some of the differences found.

p53 expression in this group of patients is found in similar percentages in lymphomas of mucosa and nodal origin, and is more frequent in Burkitt cases. The relation between p53 expression and overall survival in our series is significant, independently of histological diagnosis, and is perhaps related to treatment failure, since p53-positive patients show a sudden decrease in life expectancy during the first months after diagnosis. Multivariant regression analysis findings are in agreement with these results. They confirm that the only parameters significantly related with survival are extranodal origin, which is associated with a better prognosis, and p53 expression, which indicates a poor prognosis.

The relationship between p53 expression and low survival rates has also been found in other types of tumours (Thor et al., 1992; Visakorpi et al., 1992). This has been suggested for lymphomas (Levine et al., 1988; Cabanillas et al., 1989; Schouten et al., 1990; Rodriguez et al., 1991), based on cytogenetic studies. Significantly, Cabanillas et al. (1989) described a strikingly high rate of refractoriness to chemotherapy in patients with chromosome 17 alterations. This is similar to the early strong decrease in life expectancy for the $p53^+$ patients in our series. An association between multidrug resistance protein (MDR) and p53 protein has in fact been proposed, since mutant p53 may stimulate MDR1 promoter, and wild-type p53 could exert specific repression (Chin et al., 1992). This MDR activation could explain the absence of chemotherapeutic response in $p53^+$ patients. Recent findings about the role of p53 gene suggest new possibilities for explaining the speedier progression of $p53^+$ tumours. Different groups have shown p53 levels to increase after genotoxic injury, this high level of p53 protein being related to the capacity of cells with DNA damage to undergo apoptosis (Clarke et al., 1993; Fritsche et al., 1993; Hall et al., 1993; Lowe et al., 1993). This may imply that cells with inactivation of one or both p53 alleles (consequently lacking this mechanism of programmed cell death induction) could have a survival advantage over those without p53 alterations. The range of p53 expression detected in this series of NHLs may be a common final consequence of different ways of genetic inactivation. In fact, p53 detection by immunohistological techniques has been described as the consequence of protein stabilisation dependent on a conformational change from wild-type to mutant-type protein (Milner & Medcalf, 1991). While the wild-type p53 protein has a suppressor role in the control of the cell cycle, mutant p53 protein may have the opposite effect, inducing cell growth (Finlay et al., 1988; Hinds et al., 1989; Lane & Benchimol, 1990). Mutation of the p53 gene or conformational change in the p53 protein secondary to other causes may constitute a key step in some lymphomas, allowing further tumour expansion, as has been found in other human tumours (Sidransky et al., 1992).

The combined expression of *bcl*-2 and p53 identifies tumours with a poorer prognosis than those expressing p53 only. This is particularly significant for cases of large B-cell lymphoma presenting in lymph node. This poorer prognosis seems to be dependent on the accumulation of both *bcl*-2 and p53 expression rather than the interaction between them. However, comparative analysis between the groups of *bcl*-$2^+$, $p53^+$ lymphomas *vs* *bcl*-$2^-$, $p53^+$ is difficult because of the small number of cases included in both groups and the short follow-up of the *bcl*-$2^-$, $p53^+$ group. This prevents the detection of significant differences. A longer follow-up of a larger group of lymphoma patients could indicate the specific impact of each marker on survival.

The cumulative poor effect of both p53 and *bcl*-2 in large B-cell lymphomas, which is more significant in nodal tumours, could confirm the existence of multiple gene deregulation in non-Hodgkin's lymphoma. This would take place in a multistep pattern similar to that described in colorectal cancer (Fearon & Vogelstein, 1990) and would address the genetic mechanisms of apoptosis control and their disregulation as critical steps in the progression of tumours.

p53 expression could be tested for in NHLs, as a cheap and reproducible way of identifying patients with a poor prognosis. $p53^+$ tumours could be candidates for more intensive therapy, or different therapeutic approaches.

The work was supported by a grant from the Fondo de Investigaciones Sanitarias (FIS), Spain, and by the Leukaemia Research Fund, UK. F. Pezzella is a Leukaemia Research Fund research fellow. Thanks to Isabel Galvez, Alicia Muñoz, Margaret Jones, Helen Turley and Heather Morrison for their excellent technical assistance.

### References

AISENBERG, A.C., WILKES, B.M. & JACOBSON, J.O. (1988). The *bcl*-2 gene is rearranged in many diffuse B-cell lymphomas. *Blood*, **71**, 969-972.

BANKS, S.J., MATLASHEWISKI, G. & CRAWFORD, L. (1986). Isolation of human p53 specific monoclonal antibodies and their use in the studies of human p53 expression. *Eur. J. Biochem.*, **159**, 529-534.

CABANILLAS, F., PATHAK, S., GRANT, G., HAGEMEISTER, F.B., MCLAUGHLIN, P., SWAN, F., RODRIGUEZ, M.A., TRUJILLO, J., CORK, A., BUTLER, J.J., KATZ, R., BOURNE, S. & FREIREICH, E.J. (1989). Refractoriness to chemotherapy and poor survival related to abnormalities of chromosome 17 and 7 in lymphoma. *Am. J. Med.*, **87**, 167-172.

CESERMAN, E., CHADBURN, A., INGHIRAMI, G., GAIDANO, G. & KNOWLES, D. (1992). Structural and functional analysis of oncogenes and tumour suppressor genes in adult T-cell leukemia/lymphoma shows frequent p53 mutations. *Blood*, **80**, 3205-3216.

CHIN, K.V., UEDA, K., PASTAN, I. & GOTTESMAN, M.M. (1992). Modulation of activity of the promotor of the human MDR1 gene by *Ras* and p53. *Science*, **255**, 459-462.

CLARKE, A.R., PURDIE, C.A., HARRISON, D.J., MORRIS, R.G., BIRD, C.C., HOOPER, M.L. & WYLLIE, A.H. (1993). Thymocyte apoptosis induced by p53-dependent and independent pathways. *Nature*, **362**, 849-852.

COIFFIER, B., GISSELBRECHT, C., VOSE, J.M., TILLY, H., HERBRECHT, R., BOSLY, A. & ARMITAGE, J.O. (1991). For the groupe d'Etudes des Lymphomes agressifs. Prognostic factors in aggressive malignant lymphomas: description and validation of a prognostic index that could identify patients requiring a more intensive therapy. *J. Clin. Oncol.*, **9**, 211-219.

CORDELL, J.L., FALINI, B., ERBER, W.N., ABDULAZIZ, Z., MACDONALD, S., PULFORD, K.A.F., STEIN, H. & MASON, D.Y. (1984). Immunoenzymatic labelling of monoclonal antibodies using immune complexes of alkaline phosphatase and monoclonal anti-alkaline phosphatase (APAAP complex). *J. Histochem. Cytochem.*, **32**, 219-222.

COX, D.R. (1972). Regression models and life tables. *J. R. Stat. Soc.*, **34**, 187-220.

DOGLIONI, C., PELOSIO, P., MOMBELLO, A., SCARPA, A. & CHILOSI, M. (1991). Immunohistochemical evidence of abnormal expression of the antioncogene-encoded p53 phosphoprotein in Hodgkin's disease and CD30+ anaplastic lymphomas. *Hematol. Pathol.*, **5**, 67-73.

FANIDI, A., HARRINGTON, E.A. & EVAN, G.I. (1992). Cooperative interaction between c-*myc* and *bcl*-2 proto-oncogenes. *Nature*, **359**, 554-556.

FARRELL, P.J., ALLAN, G.J., SHANAHAN, F., VOUSDEN, K.H. & CROOK, T. (1991). p53 is frequently mutated in Burkitt's lymphoma cell lines. *EMBO J.*, **10**, 2879-2887.

FEARON, E.R. & VOGELSTEIN, B. (1990). A genetic model for colorectal tumorigenesis. *Cell*, **61**, 759-767.

FINKE, J., FRITZEN, R., TERNES, P., TRIVEDI, P., BROSS, K.J., LANGE, W., MERTELSMANN, R. & DOLKEN, G. (1992). Expression of *bcl*-2 in Burkitt's lymphoma cell lines: induction by latent Epstein-Barr virus genes. *Blood*, **80**, 459-469.

FINLAY, C.A., HINDS, P.W., TAN, T.H., ALIYAHU, D., OREN, M. & LEVIN, J. (1988). Activating mutations for transformation by p53 produce a gene produce that forms hsc70-p53 complex with an altered half life. *Mol. Cell. Biol.*, **8**, 531-539.

FRITSCHE, M., HAESSLER, C. & BRANDNER, G. (1993). Induction of nuclear accumulation of the tumor-suppressor protein p53 by DNA-damaging agents. *Oncogene*, **8**, 307-318.

GAIDANO, G., BALLERINI, P., GONG, J.Z., INGHIRAMI, G., NERI, A., NEWCOMB, E.W., MAGRATH, I.T., KNOWLES, D.M. & DALLA-FAVERA, R. (1991). p53 mutations in human lymphoid malignancies: association with Burkitt lymphoma and chronic lymphocytic leukemia. *Proc. Natl. Acad. Sci. USA*, **88**, 5413.

HALL, P.A., MCKEE, P.H., MENAGE, H.P., DOVER, R. & LANE, D.P. (1993). High levels of p53 in UV-irradiated normal human skin. *Oncogene*, **8**, 203-207.

HENDERSON, S., ROWE, M., GREGORY, C., CROOM-CARTER, D., WANG, F., LONGNECKER, R., KIEFF, E. & KICKINSON, A. (1991). Induction of *bcl*-2 expression by Epstein-Barr virus latent membrane protein 1 protects infected B cells from programmed cell death. *Cell*, **65**, 1107-1115.

HINDS, P.W., FINLAY, C.A., LEVINE, A.J. (1989). Mutation is required to activate the p53 gene for cooperation with the *ras* oncogene and transformation. *J. Virol.*, **63**, 739-746.

HOCKENBERRY, D., NUNEZ, G., MILLIMAN, C., SCHEREIBER, R.D. & KORSMEYER, S.J. (1990). *bcl*-2 is an inner mitochondrial membrane protein that blocks programmed cell death. *Nature*, **348**, 334-336.

ICHIKAWA, A., HOTTA, T., TSUSHITA, K., KINOSHITA, T., NAGAI, H., MURAKAMI, Y., HAYASHI, K. & SAITO, H. (1992). Mutations of p53 gene and their relation to disease progression in B-cell lymphoma. *Blood*, **79**, 2701-2707.

KAPLAN, E.L. & MEIER, P. (1958). Non-parametric estimation from incomplete observations. *J. Am. Stat. Assoc.*, **53**, 457-481.

LANE, D., BENCHIMOL, S. (1990). Oncogene or anti-oncogene? *Genes Dev.*, **4**, 1-3.

LENNERT, K. & FELLER, A.C. (1990). *Non-Hodgkin-Lymphome (nach der aktualisierten Kiel-Klassifikation)*. Springer: Berlin.

LEVINE, E.G., ARTHUR, D.C., FRIZZERA, G., PETERSON, B.A., HURD, D.D. & BLOOMFIELD, C.D. (1988). Cytogenetic abnormalities predict clinical outcome in non-Hodgkin lymphoma. *Ann. Intern. Med.*, **108**, 14-20.

LOWE, S.W., SCHMITT, E.M., SMITH, S.W., OSBORNE, B.A. & JACKS, T. (1993). p53 is required for radiation-induced apoptosis in mouse thymocytes. *Nature*, **362**, 847-849.

MILNER, J. & MEDCALF, E.A. (1991). Cotranslation of activated mutant p53 with wild type drives the wild-type p53 protein into the mutant conformation. *Cell*, **65**, 765-774.

PETO, R., ROE, F.J.C., LEE, P.N., LEVY, L. & CLACK, J. (1975). Cancer and ageing in mice and men. *Br. J. Cancer*, **32**, 411-426.

PEZZELLA, F., TSE, A.G.D., CORDELL, J.L., PULFORD, K.A.F., GATTER, K.C. & MASON, D.Y. (1990). Expression of the *bcl*-2 oncogene protein is not specific for the 14;18 chromosomal translocation. *Am. J. Pathol.*, **137**, 225-232.

PEZZELLA, F., JONES, M., RALFKIAER, E., ERSBOLL, J., GATTER, K.C. & MASON, D.Y. (1992). Evaluation of *bcl*-2 protein expression and 14;18 translocation prognostic markers in follicular lymphoma. *Br. J. Cancer*, **65**, 87-89.

PEZZELLA, F., MORRISON, H., JONES, M., GATTER, K.C., LANE, D., HARRIS, A.L. & MASON, D.Y. (1993). Immunohistochemical detection of p53 and *bcl*-2 protein in non-Hodgkin's lymphoma. *Histopathology*, **22**, 39-44.

RAGHOEBIER, S., KRAMER, M.H.H., VAN KRIEKEN, J.H.J.M., DE JONG, D., LIMPENS, J., KLUIN-NELEMANS, J.C., VAN OMMEN, G.J.B. & KLUIN, PH. M. (1991). Essential differences in oncogene involvement between primary nodal and extranodal large cell lymphoma. *Blood*, **78**, 2680-2685.

RODRIGUEZ, M.A., FORD, R.J., GOODACRE, A., SELVANAYAGAM, P., CABANILLAS, F. & DEISSEROTH, A.B. (1991). Chromsome 17p and p53 changes in lymphoma. *Br. J. Haematol.*, **79**, 575-582.

SCHOUTEN, H.C., SANGER, W.G., WEISENBURGER, D.D., ANDERSON, J. & ARMITAGE, J.O. (1990). Chromosomal abnormalities in untreated patients with non-Hodgkin's lymphoma: associations with histology, clinical characteristics, and treatment outcome. *Blood*, **75**, 1841-1847.

SETO, M., JAEGER, U., HOCKETT, R.D., GRANINGER, W., BENNETT, S., GOLDMAN, P. & KORSMEYER, S.J. (1988). Alternative promoters and exons, somatic mutation and deregulation of the *bcl*-2-Ig fusion gene in lymphoma. *EMBO J.*, **7**, 123-131.

SIDRANSKY, D., MIKKELSEN, T., SCHWECHHEIMER, K., ROSENBLUM, M.L., CAVANEE, W. & VOGELSTEIN, B. (1992). Clonal expansion of p53 mutant cells is associated with brain tumour progression. *Nature*, **355**, 846-847.

THOR, A.D., MOORE, D.H., EDGERTON, S.M., KAWAASAKI, E.S., REIHSAUS, E., LYNCH, H.T., MARCUS, J.N., SCHWARTZ, L., CHEN, L.L., MAYALL, B.H. & SMITH, H.S. (1992). Accumulation of p53 tumour suppressor gene protein: an independent marker of prognosis in breast cancers. *J. Natl Cancer Inst.*, **84**, 845-855.

VELASQUEZ, W.S., JAGANNATH, S., TUCKER, S.L., FULLER, L.M., NORTH, L.B., REDMAN, J.R., SWAN, F., HAGEMEISTER, F.B., MCLAUGHLIN, P. & CABANILLAS, F. (1989). Risk classification as the basis for clinical staging of diffuse large cell lymphoma derived from 10-year survival data. *Blood*, **74**, 551-557.

VILLUENDAS, R., PIRIS, M.A., ORRADRE, J.L., MOLLEJO, M., RODRIGUEZ, R. & MORENTE, M. (1991). Different *bcl*-2 protein expression in high-grade B-cell lymphomas derived from lymph node or mucosa-associated lymphoid tissue. *Am. J. Pathol.*, **139**, 989-993.

VILLUENDAS, R., PIRIS, M.A., ORRADRE, J.L., MOLLEJO, M., ALGARA, P., SANCHEZ, L., MARTINEZ, J.C. & MARTINEZ, P. (1992). p53 protein expression in lymphomas and reactive lymphoid tissue. *J. Pathol.*, **166**, 235-241.

VISAKORPI, T., KALLIONEMI, O.P., HEIKKINEN, A., KOIVULA, T. & ISOLA, J. (1992). Small subgroup of aggressive, highly proliferative prostatic carcinomas defined by p53 accumulation. *J. Natl Cancer Inst.*, **84**, 883-887.

WIMAN, K.G., MAGNUSSON, K.P., RAMQUIST, T. & KLEIN, G. (1991). Mutant p53 detected in a majority of Burkitt lymphoma cell lines by monoclonal antibody PAb240. *Oncogene*, **6**, 1633-1639.

YUNIS, J.J., MAYER, M.G., ARNESEN, M.A., AEPPLI, D.P., OKEN, M.M. & FRIZZERA, G. (1989). *bcl*-2 and other genomic alterations in the prognosis of large-cell lymphoma. *N. Engl. J. Med.*, **320**, 1047-1054.

ZUTTER, M., HOCKENBERRY, D., SILVERMAN, G.A. & KORSMEYER, S.J. (1991). Immunolocalization of the *bcl*-2 protein within haematopoietic neoplasms. *Blood*, **78**, 1062-1068.