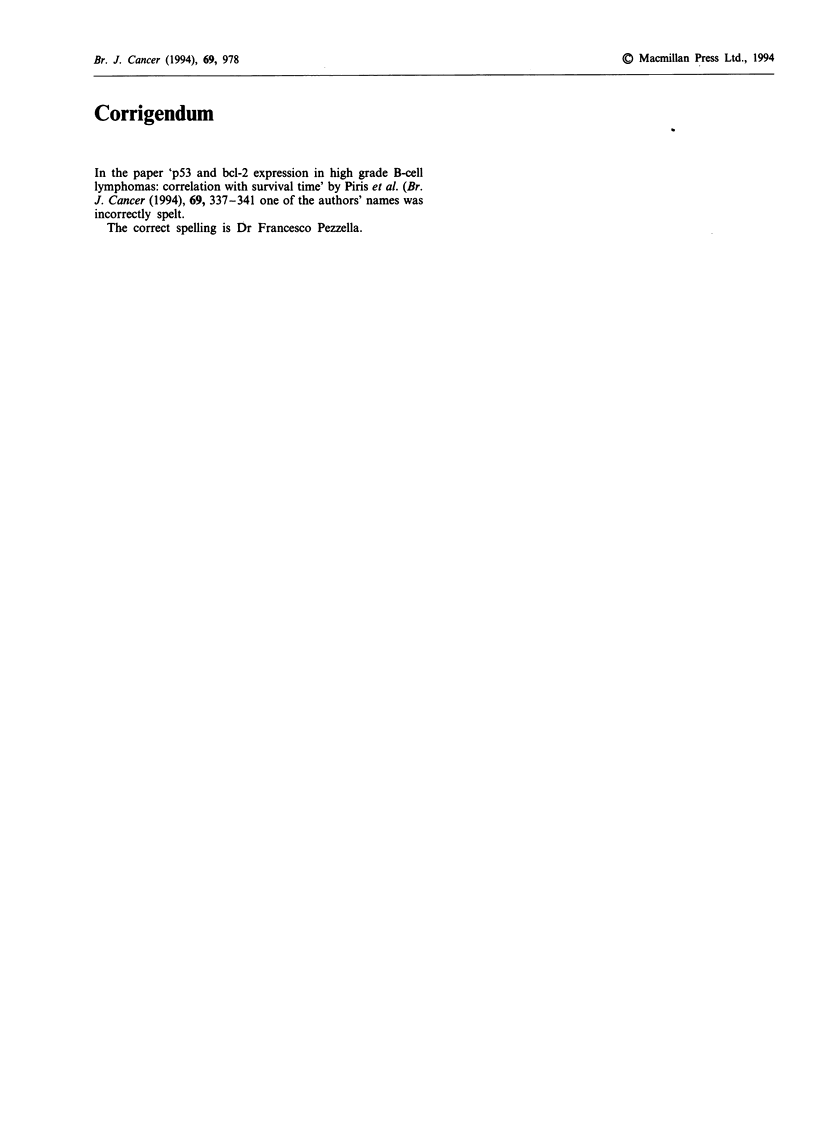# Corrigendum

**Published:** 1994-05

**Authors:** 


*Br. J. Cancer* (1994), **69**, 978 © Macmillan Press Ltd., 1994

### Corrigendum

In the paper 'p53 and bcl-2 expression in high grade B-cell lymphomas: correlation with survival time' by Piris *et al.* (*Br. J. Cancer* (1994), **69**, 337–341 one of the authors' names was incorrectly spelt.

The correct spelling is Dr Francesco Pezzella.